# Robust Arm and Hand Tracking by Unsupervised Context Learning

**DOI:** 10.3390/s140712023

**Published:** 2014-07-07

**Authors:** Vincent Spruyt, Alessandro Ledda, Wilfried Philips

**Affiliations:** 1 Cosys lab, Antwerp University, Paardenmarkt 92, 2000 Antwerp, Belgium; E-Mail: ledda.alessandro@yahoo.com; 2 Image Processing and Interpretation, iMinds, Ghent University, St-Pietersnieuwstraat 41, 9000 Ghent, Belgium; E-Mail: philips@telin.UGent.be

**Keywords:** hand tracking, particle filter, unsupervised learning, random forest, context learning, importance sampling

## Abstract

Hand tracking in video is an increasingly popular research field due to the rise of novel human-computer interaction methods. However, robust and real-time hand tracking in unconstrained environments remains a challenging task due to the high number of degrees of freedom and the non-rigid character of the human hand. In this paper, we propose an unsupervised method to automatically learn the context in which a hand is embedded. This context includes the arm and any other object that coherently moves along with the hand. We introduce two novel methods to incorporate this context information into a probabilistic tracking framework, and introduce a simple yet effective solution to estimate the position of the arm. Finally, we show that our method greatly increases robustness against occlusion and cluttered background, without degrading tracking performance if no contextual information is available. The proposed real-time algorithm is shown to outperform the current state-of-the-art by evaluating it on three publicly available video datasets. Furthermore, a novel dataset is created and made publicly available for the research community.

## 1. Introduction

Robustly tracking human hands in real-time, through cluttered scenes with changing illumination, is one of the most challenging tasks in current HCI (Human-Computer-Interaction) research. Whereas the task of tracking rigid objects with a known appearance has been largely solved in academic literature, tracking non-rigid objects remains a difficult problem. Specifically, the appearance of human hands greatly depends on their pose and can not be easily described using discriminative features. Hands are non-rigid, highly articulated objects with a large number of degrees of freedom: 27 in total, six of which represent translation and rotation of the wrist [1].

To reduce the dimensionality of the problem, a common approach is to resort to template based tracking and detection methods that can track hands in predefined poses only [2–5]. In many real-life applications however, the user's behaviour should be restricted as little as possible. Consequently, these methods have limited applicability in practical applications. On the other hand, tracking methods that can cope with non-rigid hand poses usually resort to a (skin-)color based tracking approach due to the lack of other discriminative and easily exploitable features [6–14]. However, these methods tend to fail in unconstrained environments with changing background and illumination.

In [15,16] we proposed to combine a particle filter based tracking framework with a discriminative classifier to overcome these difficulties. By combining different cues such as skin-color probability, motion likelihood and optical flow estimates into a probabilistic framework using a sensor-fusion approach, local maxima in the likelihood function can be overcome. We showed that our method can track hands robustly in unconstrained environments and greatly outperforms the state-of-the-art solutions. However, a major shortcoming of the proposed technique is its tendency to be easily distracted when the hand occludes the face, another hand, or skin-colored background objects that exhibit a hand-like visual appearance.

In this paper we formulate a solution to the above problem, based on the idea that a moving object is usually embedded in a specific context and rarely moves alone. In the case of human hands, an obvious example of such contextual link would be the arm which exhibits a motion pattern that is strongly correlated to that of the attached hand. Although an arm detector could be trained offline based on color cues [17], edge detection [18] or background subtraction [19], these methods tend to fail in unconstrained environments where the appearance of the subject's arm is unknown before tracking starts. Instead, we propose a method to exploit temporal correlation, in order to automatically learn which objects appear to be temporally connected to the hand. These objects can then be used to increase tracking performance in case of occlusion of the hand itself.

Furthermore, the proposed solution is not limited to arm tracking. In many practical applications, the tracked hand holds an object such as a smartphone or game controller, or attributes such as an umbrella (*i.e.*, surveillance applications). Whereas this degrades tracking performance if a traditional hand tracking algorithm were to be used [16], it can actually improve tracking if the system is able to learn that the object's position is temporally correlated with the hand.

**Figure 1.**
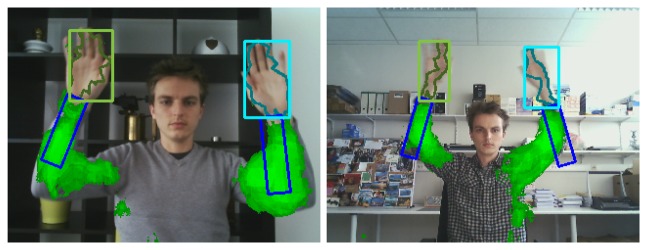
Illustration of contextual learning. Objects colored in green exhibit a temporally correlated behaviour to the hands, and are learned automatically. Hand and arm bounding boxes are estimated by partitioned sampling particle filtering, and the hand segmentation mask is obtained by active contour modeling.

Apart from increasing tracking robustness, our temporal learning scheme automatically results in a rough segmentation of the supporting object (e.g., human arm). This could be of interest in many applications such as surveillance or human-computer-interaction. Figure 1 illustrates this by drawing the supporting pixels in green, where the intensity of the color indicates the probability of correct classification. The segmentation mask shown by Figure 1 and all following figures was obtained by means of a simple and efficient active contour segmentation of the likelihood image described in [16]. The internal energy was set to zero, and smoothing is imposed by a postprocessing step on the resulting contour. The segmentation mask does not directly contribute to the context learning method described in this paper and is only shown for visualisation purposes.

It is important to note that the context segmentation, shown in green, is obtained using a single, static video frame, and is not the result of motion detection. Therefore, our method is able to detect and segment the supporting objects even in video frames that do not contain any motion.

We introduce a technique to automatically learn this context in real-time. Based on this continuous learning process, we propose two methods to increase object tracking performance. The first method models the spatial relationship between context and object of interest, and incorporates this information into the proposal distribution of a particle filter. Therefore, this method naturally extends to object tracking in general. The second method extends the state space of the hand tracker with an extra parameter representing the arm and uses a context-based observation model to obtain the likelihood estimates. This not only yields an estimate of the arm location, but also increases tracking robustness in case of occlusion.

The remainder of this paper is outlined as follows: In Section 2 we briefly discuss related work from literature. Sections 3.1 and 3.2 provide an extensive discussion of the online learning process. In Section 3.3 we discuss how the contextual information is incorporated into the proposal distribution of a particle filter to improve tracking robustness, and in Section 3.4 we propose an efficient method to track the arm together with the corresponding hand. Finally, in Section 4 we evaluate our algorithm by comparing the results with state-of-the-art solutions using three publicly available datasets.

## 2. Related Work

Learning the context of an object of interest is known to increase detection performance of object detectors. For instance, Mittal *et al.* [20] explicitly train a parts based deformable model to capture the arm's appearance while designing a hand detector. Their evaluation shows that including contextual information yields a 10% increase in recall.

Similarly, Torralba [21] proposes to use a statistical model of low-level features to obtain a prior which is used to improve object detection rates. However, since object detectors are trained offline and should be able to detect objects embedded in an unknown context, only statistically significant relations between the context of the training data and the objects of interest can be learned. In contrast, object trackers are embedded in one specific context and could learn this context online. Furthermore, tracking can benefit from learning temporary links between the context and the tracked object. These links might appear and disappear at any time but can greatly increase robustness against occlusion and background clutter when available.

Therefore, our goal is to automatically learn a model of the current context that supports the object of interest in an unsupervised manner, based on few and unreliable measurements. The model should adapt online and should be able to quickly learn and forget new information without overfitting.

The idea of unsupervised learning of unlabeled data was explored by Kalal *et al.* [22,23], resulting in the well known “predator” tracking algorithm. Kalal *et al.* introduce the concept of structured unlabeled data which is based on the observation that the labels of data in computer vision often exhibit strong spatio-temporal dependencies. Structured unlabeled data is then defined as unlabeled data for which the label is restricted if the label of related data is known. In a video sequence, the location of an object defines a trajectory in time. Unlabeled image patches close to this trajectory are likely to be samples from the same object class and are therefore considered structured data that can be used to train an online classifier.

**Figure 2.**
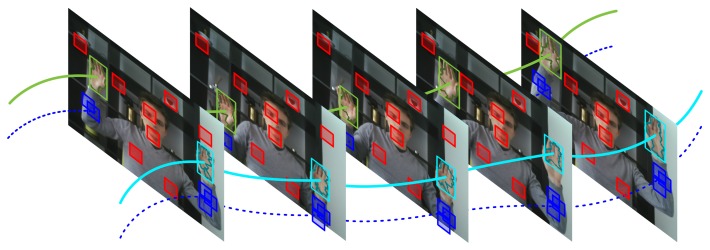
Structured data, shown in red and blue, is defined by the known hand trajectories shown in cyan and green. Blue patches exhibit motion that is correlated to the motion of the labeled hands, whereas red patches are negative samples.

However, although the predator algorithm is able to quickly learn an appearance model for the object of interest, it was shown to fail when applied to the task of hand tracking [15]. The main reason for this is that the appearance of hands varies widely throughout the video sequence, making it difficult to learn an accurate model. In this paper, we therefore extend the concept of structured unlabeled data by considering trajectories of other objects in the scene that show a temporal correlation to the trajectory of the object of interest. Instead of trying to learn the appearance of the object of interest, we propose to learn the appearance of surrounding objects that move coherently with the object of interest, as illustrated by Figure 2. These surrounding objects can then be used to steer the tracking algorithm in case of uncertainty due to occlusion or cluttered backgrounds.

A related idea was explored by Cerman *et al.* [24]. In their work, objects are tracked using an affine system model. A foreground model representing all coherently moving objects is learned online. Although their approach illustrates that contextual learning can greatly improve tracking performance, this method takes approximately 10 seconds to process a single video frame on modern hardware. Furthermore, a background model is learned offline by processing the complete video before actual tracking starts. Therefore, this solution is not suited for real-time tracking applications.

Grabner *et al.* [25] extended the work of Cerman *et al.* by defining so called “supporters” instead of trying to obtain a complete model of the foreground and background. Supporters are regions in the image that seem to move coherently with the object of interest. An online learned implicit shape model is used to describe the object of interest and its supporting regions by means of SIFT descriptors. A generalized Hough transform is then employed such that each supporting region votes for the object's position. However due to the use of the implicit shape model and several computationally expensive feature descriptors, this approach can not be used for real-time purposes. Furthermore, if supporting regions are anisotropic they can often not be used to obtain an estimate of the object's location. An obvious example are regions on a human arm which are not discriminative in the tangential direction. Finally, the method proposed by Grabner *et al.* simply performs tracking by detection in each video frame and therefore does not fully exploit temporal consistency.

Inspired by the work of Grabner *et al.*, we propose an online learning scheme to model image regions that move coherently with the hand, and we incorporate this information probabilistically in a particle filter framework. Our solution operates in real-time and as a side-effect also produces a rough segmentation of the supporting objects. Supporting regions provide an estimate of the object's location, which is used to steer the particle filter. We propose a novel proposal distribution that greatly increases sampling efficiency, resulting in robust tracking with only a few particles to represent the posterior distribution of the state estimate.

## 3. Materials and Methods

Online contextual learning is a continuous process, such that the tracking algorithm becomes more robust as time progresses. At each time instance, parts in the scene that are close to and move coherently with the hand are use to train a classifier in an online manner. Both the process of selecting these training samples, and the classifier that is trained based on these samples, are discussed in the next sections.

At each time instance, every pixel in the image is labeled by this classifier as being either part of the context, or part of the background. Pixels that are part of the hand's context are then used to steer the particle filter, thereby increasing its robustness against occlusion and cluttered backgrounds. In the next sections, we propose two methods to incorporate contextual information into a probabilistic tracking scheme. Whereas the first method can be generally applied to any object tracking problem, the second method is specifically focussed on hand-tracking, and involves estimating the arm's position.

### 3.1. Image Patch Selection Using Structural Constraints

The remainder of this paper is based on the hand detection and tracking algorithm that we proposed earlier in [15,16]. This method was shown to be able to robustly track hands in unconstrained environments, and to automatically initialize and recover from errors.

Given the hand location and trajectory, we define positive image patches as those patches that appear to move coherently with the tracked hand, whereas negative patches are defined as those image regions that do not exhibit such correlation. This is illustrated by Figure 2 in which positive patches are shown in blue, while randomly sampled negative patches are indicated by red rectangles.

We define positive image patches as those patches that are close to the trajectory of the tracked hands. These patches are therefore defined by spatio-temporal constraints. Only those patches within a neighborhood of the hand that move coherently with the hand are considered positive. The labels of patches within this neighborhood that do not exhibit coherent movement, and the labels of patches outside this neighborhood that do move coherently with the hand, are considered unknown. Finally, patches outside this neighborhood that do not move coherently with the hand, are labelled as negative patches. This concept of using structured data for unsupervised learning was introduced by Kalal *et al.* [22,23] and is known as PN-learning. Kalal *et al.* showed formally that iterative PN-learning can converge to a zero-error classifier if certain assumptions are met such that errors that are introduced by incorrectly labeled positive patches and incorrectly labeled negative patches cancel out each other. Although this assumption does not necessarily hold in practice, it was shown that PN-learning using imperfect constraints greatly boosts classification performance.

Since we use spatio-temporal constraints to exploit the structural nature of the unlabeled data, training data is only available at time instances where the tracked hands actually move. To obtain a measure of motion coherency, the real-time, sparse optical flow algorithm that we proposed earlier in [26] is used. This method yields a sparse, regularized flow-field and was used to steer the motion model of the hand tracking particle filter in [15,16]. Based on the optical flow estimates, positive samples can easily be defined as those samples in a neighborhood of the hand, whose optical flow vector is similar to the average of all flow vectors within the hand region itself.

In the following let **x** = (*x*, *y*) be a position in the image, around which we consider a square region with scale *s*, such that the width of the region is 2*s*. We denote these regions by *R_s_*_,**x**_. Let **f**(**x**) = (*u*, *v*) be the optical flow vector that represents the velocity and direction of the motion of this region from the previous frame to the current frame. Finally, let **h** = (*x*, *y*) represent the position of the hand in the video frame, and let **f̄**(**h**) represent the average flow within the bounding box of the tracked hand.

A spatial constraint can then be defined simply by considering only patches for which *ϵ* = ‖**x** − **h**‖_2_ < *τ* , where *τ* is a parameter that defines the size of the neighborhood. Similarly, the temporal constraint is defined simply by *θ* = ‖ **f**(**x**) − **f̄**(**h**) ‖ < *δ*, where *δ* is a parameter that defines the temporal coherency of two optical flow vectors.

Each image patch *R_s_*_,**x**_ can then be assigned a label *l*(*R_s_*_,**x**_) ∈ {−1, 0, 1}, where *l* = −1 represents the fact that no informed decision can be made, whereas *l* =0 represents a background patch and *l* = 1 represents a patch that moves coherently with the hand. Image patches are labeled as follows:

(1) $$l(R_{s,\text{x}})=\{\begin{array}{ll} 0 & \text{if}\;\epsilon \geq \tau \;\text{and}\;\theta \geq \delta \\ -1 & \text{if}\;\epsilon \;\geq \tau \;\text{and}\;\theta <\delta \\ 1 & \text{if}\;\epsilon \;<\tau \;\text{and}\;\theta <\delta \\ -1 & \text{if}\;\epsilon <\tau \;\text{and}\;\theta \geq \delta \end{array}$$

The scale s of the square image patches at time instance *t* is chosen such that 2*s* = min(**r**), where **r** =(*w*, *h*) represents the width and height of the hand in that video frame. Dynamic scale selection results in a more efficient usage of the available training data and increases scale invariance of the final classifier.

Although real-time, dense optical flow algorithms exist [27,28], in the context of our particle filter framework the optical flow estimation stage should only consume a fraction of the available CPU power. The sparse optical flow estimation method used in this paper was proposed in [26] and only needs 6 ms of processing time on QVGA video. Similar to most dense optical flow algorithms, this method could easily benefit from GPU parallelization to decrease processing times even further. Due to the nature of the sparse optical flow algorithm used, optical flow vectors are often found around the edges of an object. As a result, positive regions centered at those edge points tend to contain a lot of background pixels, slowing down the learning process. Therefore, we propose to slightly relocate each positive image patch such that it contains as much foreground pixels as possible. Assuming the foreground contains moving pixels while the background consists of static objects, this corresponds to shifting the region's centroid such that the amount of moving pixels within the region is maximized. Since training data is only gathered if the hands are moving, the first assumption is valid per definition. The second assumption does not necessarily hold. However, in case both the background and foreground contain moving pixels, the current position of the positive region already maximizes the amount of motion within the bounding box such that no new position will be found. Therefore, in the worst case the region is not moved at all, whereas the best case scenario moves the region closer towards the center of the supporting object.

For performance reasons we simply detect moving pixels by temporal frame differencing. Since the frame difference image *T* is also used by the particle filter framework during hand tracking [15], no additional computational cost is incurred. Each positive region is then pushed towards its closest local maximum within the frame difference image *T* by executing a mean-shift iteration:

(2) $$\hat{\text{x}}=\text{x}+\Delta \text{x}\;:\Delta \text{x}=\frac{\underset{\text{p}\in W}{\text{∑}}K(\text{p}-\text{x})T(\text{p})(\text{p}-\text{x})}{\underset{\text{p}\in W}{\text{∑}}K(\text{p}-\text{x})T(\text{p})}$$

where *K*(.) defines a smoothing kernel and *W* represents the set of pixels within a window surrounding **x**. We use a simple uniform kernel, the bandwidth of which is defined by the scale *s* of the region under consideration. This allows us to re-use the integral images that are also used by the hand tracking particle filter, to execute the mean-shift iteration in constant time.

**Figure 3.**
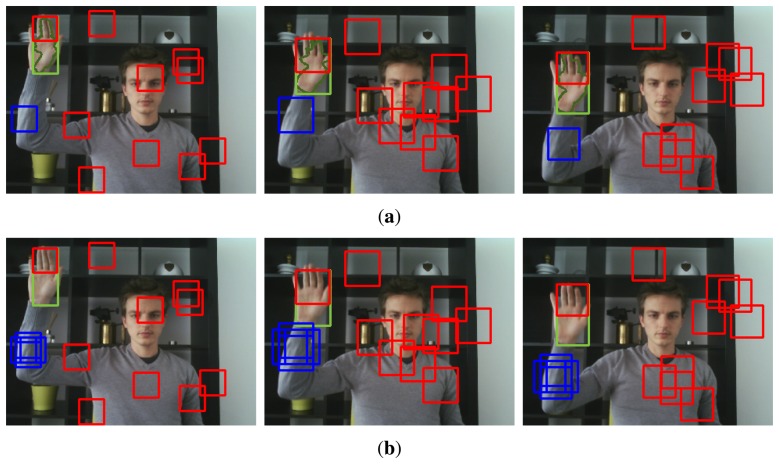
Positive (context) and negative (background) patches are sampled automatically to train an online Random Forest classifier. Positive samples are shown in blue, whereas negative samples are shown in red. (**a**) Positive and negative patch candidates are selected automatically, based on the optical flow estimates. (**b**) Positive patches are pushed towards the center of the arm and slightly shifted copies are instantiated.

Finally, several slightly shifted versions of each region are added to the set of positive image patches, in order to increase translational invariance of the final classifier. Figure 3 illustrates the PN-learning process graphically. The top-row shows the positive (*l*(*R_s_*_,**x**_) = 1) and negative (*l*(*R_s_*_,**x**_) = 0) patch candidates respectively by blue and red rectangles. The bottom-row shows the positive patch candidates after mean-shift iteration and the introduction of small spatial shifting.

Each region *R_s_*_,**x**_ is described using several simple feature descriptors. Although robust spatial descriptors such as Histograms of Oriented Gradients (HOG) [29], SIFT [30] and SURF [31] or spatio-temporal descriptors as discussed in [32] could be used to further improve our detection rates, these descriptors introduce a high computational complexity. Therefore, to allow real-time processing, we use several simple feature descriptors to model the color and texture of each image patch.

Color is represented simply by three 16-bin histograms. To reduce the computational complexity of our solution, we first calculate the integral histograms after which the histogram of each arbitrary region in the image can be obtained in constant time. Similar to the well known integral images, proposed by Viola and Jones [33], an integral histogram is a recursive propagation method to calculate a cumulative histogram for each pixel in the image, by means of dynamic programming. At each pixel location, this cumulative histogram is the histogram of all pixels above and to the left of the pixel under consideration. For each pixel location an integral histogram, represented by vectors of integers and with bin size 16, is obtained using a single pass propagation method during a preprocessing phase.

Although color can be a discriminative cue, it is inherently sensitive to illumination changes and can not represent local structure. Therefore, we model fine texture details by a 16-bin local binary pattern (LBP) [34] histogram. The local binary pattern was originally defined by Ojala *et al.* [34] as an ordered set of binary comparisons between a center pixel and each of its surrounding pixels, and was shown to have high discriminative power for texture classification problems. The LBPs are calculated for each pixel in the image, after which the histogram of LBPs within a region can be used to describe the texture of this region. However, since each pixel is surrounded by eight neighbors, an 8-bit LBP descriptor results in a 256-bin histogram, many of which are empty if small regions have to be described. Therefore, to avoid the well known “curse of dimensionality”, we propose to use the Center Symmetric Local Binary Pattern (CS-LBP) operator [35], which has been proven to outperform both the original LBP and the well known SIFT descriptor in various image recognition problems. The CS-LBP is simply obtained by using the sign of the difference between center-symmetric pairs of pixels, instead of the difference between each pixel and a central pixel, resulting in a 4-bit descriptor and thus a 16-bin histogram. The CS-LBP can be implemented efficiently using only bit-shifting operations.

While the LBP descriptor models fine grained texture details, we use several Haar-like features [33] to model larger structural properties of the image patch. Haar-like features are defined as the normalized difference between distinct subregions of the image patch under consideration and can therefore model larger structural properties of the object. Haar-like features can be calculated efficiently and in constant time by first obtaining the so called integral image, similar to the concept of integral histograms as was explained above.

Finally, these Haar-like features are not only calculated using the grayscale version of the video frame, but also based on a skin probability map in which each pixel contains the probability of it representing human skin. We used the publicly available Compaq dataset [36] to train a Bayesian skin classifier. The Compaq dataset contains over 3000 manually segmented skin coloured images and over 4000 non-skin coloured images. During training of the skin classifier, we simply model the likelihood *P*(*r*, *g*, *b*|*skin*) of observing a certain RGB triplet in a skin region by means of a 32-bin 3D histogram of skin pixels. Similarly, the likelihood *P*(*r*, *g*, *b*|¬*skin*) of observing this color in a non-skin region, is modeled by means of a 32-bin 3D histogram of non-skin pixels. Furthermore, the prior probabilities *P*(*skin*) and *P*(¬*skin*) =1 − *P*(*skin*), can be obtained directly from the training data. The posterior probability *P*(*skin*|*r*, *g*, *b*) can then be obtained according to Bayes' rule:

$$\begin{array}{ll} P(\text{skin}|r,g,b) & =\frac{P(r,g,b|\text{skin})P(\text{skin})}{P(r,g,b)}\\ & =\frac{P(r,g,b|\text{skin})P(\text{skin})}{P(r,g,b|\text{skin})P(\text{skin})+P(r,g,b|\neg \text{skin})P(\neg \text{skin})} \end{array}$$

**Figure 4.**
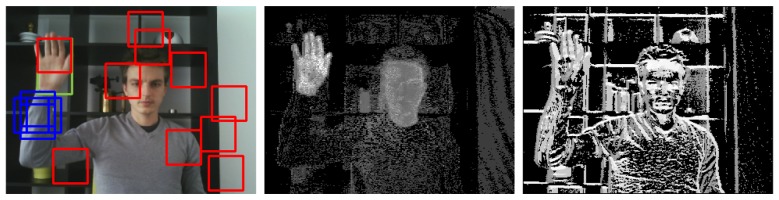
The original image, the skin probability map and the CS-LBP image.

Figure 4 shows the skin probability map and the CS-LBP image together with the original video frame for illustrative purposes.

### 3.2. Robust Online Learning and Forgetting

Since the training data becomes available only incrementally as time progresses, an online learning scheme is needed. Furthermore, the chosen classifier needs to be extremely robust to noise in the training data, since it is expected that errors occur during the image patch selection process described in the previous paragraph. Finally, the classifier should be able to automatically select the most discriminative features from the set of available features, and should be able to forget outdated information that was learned in the past.

A well known issue that should be considered when choosing a classifier and that governs many important requirements such as robustness to noise and overfitting, is the bias-variance trade-off. The bias of a classifier corresponds to the systematic errors made by the classifier, independently on the training subset. The decision boundaries of a linear classifier for instance are determined by only a few parameters, such that approximately the same boundaries would be obtained if the classifier were to be trained on different subsets of the training population. Therefore, linear classifiers would consistently miss-classify the same samples across its training sets, and are known to be high-bias classifiers. On the other hand, classifiers such as decision trees, neural networks or K-nearest-neighbour yield decision boundaries that closely fit the training data and are therefore low-bias classifiers.

Whereas a low-bias classifier might seem preferable, this is not necessarily always the case. A classifier that has a low average error, and is thus low-bias, might still make a lot of errors when trained on one data subset, whereas only few errors are made when trained on another subset. This variability is called classifier variance, and is a second important measure of classification performance. A linear classifier, for instance, is a low-variance classifier, since the number of miss-classifications would be stable across chosen training sets. Decision trees, neural networks or K-nearest-neighbour classifiers on the other hand, are high-variance classifiers, since the number of miss-classifications strongly depends on the chosen training set.

In general, classifiers that are able to fit the data well and often tend to overfit, exhibit low bias but high variance, while classifiers that result in more general decision boundaries yield high bias but low variance. Although a low-bias, low-variance classifier is desirable, lowering the variance of a classifier, often implicitly increases the bias, and vice versa. If a low-bias classifier, such as a decision tree, is available, an obvious way to lower the variance, is to train multiple decision trees on different subsets of the population, and then use the average decision (*i.e.*, regression) or a voting scheme (*i.e.*, classification) as the final result. However, in practice only a limited amount of training data is available, instead of the whole population. A well known method to approximate the distribution of the complete population if only a limited number of observations are available, is to construct multiple training samples by bootstrapping the original training dataset. Bootstrapping is a resampling method that is known by statisticians as sampling with replacement.

The concept of aggregating votes of classifiers that are trained on bootstrapped samples of the training data is called *b*ootstrap *agg*regating or *bagg*ing [37] and is a well known technique to obtain a low-variance ensemble classifier. However, bagging generally increases the bias of a classifier due to the high correlation between each bootstrapped classifier as a result of the duplicated training samples. To further lower the bias of the ensemble classifier, each classifier of the ensemble could be trained on a different subset of features, thereby reducing their correlation. The idea of combining bagging with random feature selection is used by random forest classifiers [38].

A random forest is an ensemble of decision tree classifiers, each of which are trained using a bootstrapped sample of the original training data. Furthermore, each decision tree is trained by randomly selecting a subset of the available feature descriptors, and then using those features that best split the data based on the class labels. Random forests therefore are low-variance, low-bias classifiers that have been shown to be extremely resilient to overfitting problems. Furthermore, bagging greatly increases the classifier's robustness to noise. Breiman [38] showed that randomly changing a fraction of the labels of the training data causes a decrease in classifier performance when using traditional boosting techniques, whereas bagging seems to be immune to the introduced noise. Finally, random forest classifiers can be implemented efficiently as a sequence of conditional statements and are therefore perfectly suited for real-time applications.

Saffari *et al.* [39] recently reformulated the problem of training random forests to an incremental learning process using an online decision tree growing procedure. Furthermore, their method allows for a temporal weighting scheme to automatically discard old and irrelevant trees while adding new trees to the ensemble when needed. Saffari *et al.* further showed that the performance of the online random forest algorithm converges to that of an offline trained random forest.

In this paper, we therefore use the online random forest algorithm proposed by Saffari *et al.* to dynamically learn a representation of the supporting regions of the tracked hand. In our experiments we use 10 decision trees which are grown to a maximum depth of 20. Furthermore, 200 random tests are executed during each feature selection iteration while growing the trees.

**Figure 5.**
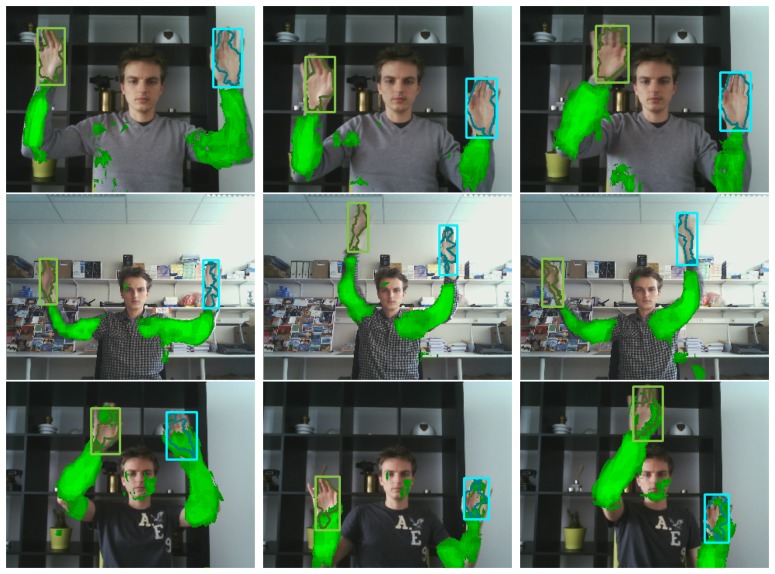
Pixels that are classified as being part of the hand's context are shown in green. The brightness indicates the posterior probability (classifiers certainty) that the label assignment was correct.

Figure 5 shows several classification examples. The center pixel of each region *R_s_*_,**x**_ is indicated in green if classified as being part of the hand's context. The brightness represents the posterior probability of being part of this context and thus indicates the classifier's certainty.

**Figure 6.**
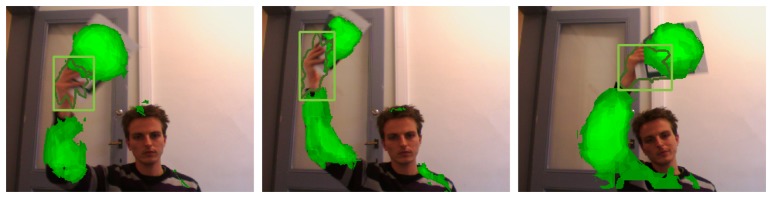
Context learning allows our hand tracker to automatically learn the appearance of any object that exhibits coherent motion with the hand itself. In this image, the person is holding a magazine.

Furthermore, although we are mostly interested in learning the appearance of the arm, due to our related research on hand tracking, the proposed context learning scheme can also be used to learn the appearance of other attributes. This is illustrated by Figure 6, in which the person holds a magazine. The classifier learns that the magazine exhibits coherent motion with the hand and therefore learns its appearance in real-time.

### 3.3. A Context Based Proposal Distribution for Particle Filtering

The random forest classifier yields a probabilistic estimate about the class label of each pixel in the image. In Figure 6, these probabilities for the positive class labels are represented by the intensity of the green overlay. This set of probabilities therefore represents a spatial distribution. Throughout the video frame, the modes of this distribution will shift due to the motion of the arm or the supporting objects.

**Figure 7.**
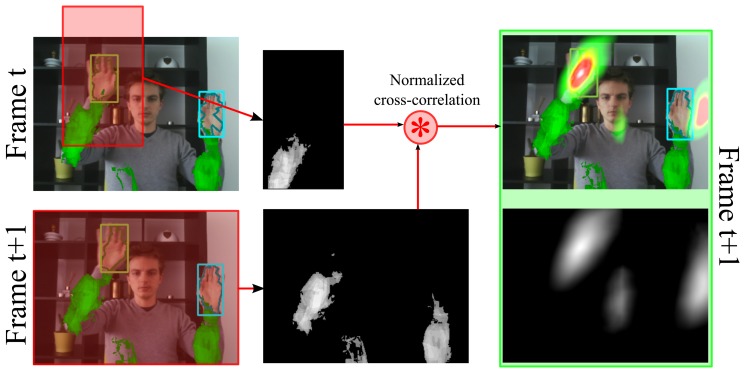
In this illustration, the location of the left hand is estimated in frame t + 1, based on its context in frame t. Cross-correlation based template matching is used to match a region of interest in the context classification distribution of frame t, with the context classification distribution of frame t + 1. The result is a prediction of the hand location, purely based on static, contextual information.

Therefore, instead of tracking the hand directly, we can track the changes in the context classification distribution around the hand, and use these changes to predict the location of the hand in the next video frame. Although robust histogram comparison methods, such as the Earth Mover's Distance, are available, many of these have a high computational complexity. Instead, we propose to simply use the normalized cross-correlation as a measure of correspondence between the spatial context-histograms of subsequent frames, since this can be implemented efficiently using integral images [40]. On a side node, normalized cross-correlation is often used for template matching. Therefore, our approach of finding the hand in the next frame could also be interpreted as a template matching solution, where the template is defined by the context classification result of the previous frame. This is illustrated more clearly by Figure 7, in which the position of the left hand is estimated.

Normalized cross-correlation is defined as

(3) $$r(u,v)=\frac{\sum_{x,y}(f(x,y)-{\bar{f}}_{u,v})(t(x-u,y-v)-\bar{t})}{\sqrt{\left(\sum_{x,y}{\left(f(x,y)-{\bar{f}}_{u,v}\right)}^{2}\sum_{x,y}{\left(t(x-u,y-v)-\bar{t}\right)}^{2}\right)}}$$

where *f*(*x*, *y*) represents the image under consideration, *t*(*x*, *y*) is the template, *f̄_u_*_,_*_v_* represents the mean of *f*(*x*, *y*) in the region of the template centered at (*u*, *v*), and *t̄* is the mean of the template image *t*(*x*, *y*).

To obtain an estimate of the hand location we simply want to maximize this correlation. Therefore, the estimate **ŷ** = (*x̂*, *ŷ*) is obtained as

(4) $$\hat{\text{y}}=\underset{\text{y}}{\arg\max}r\left(\text{y}\right)$$

This state estimate is complementary to the skin-color and motion based state estimate as obtained by the hand tracker used [16]. The question now remains how to incorporate this estimate into the particle filter framework to improve tracking performance. The skin-color and motion based likelihood, used by the hand tracker's observation model [15], represents $p\left({\text{y}}_{t}|{\text{x}}_{t}^{i}\right)$, where **y** is the latest observation and **x** is the state to be estimated, *i.e.*, the hand location hypothesis of particle *i*. In earlier work, we showed that this likelihood function is extremely accurate if a good estimate ${\text{x}}_{t}^{i}$ is available. Therefore, instead of incorporating the contextual information in the observation model of the particle filter, we propose to use it to improve the estimate ${\text{x}}_{t}^{i}$ of each particle, which is then simply assigned a skin-color and motion based likelihood.

One way of improving the estimate ${\text{x}}_{t}^{i}$ would be to adapt the motion model of the particle filter. Several approaches can be found in literature that incorporate the latest measurements directly into the motion model of a particle filter [41,42]. However, from a Bayesian perspective, the motion model of the particle filter, yielding the state transition prior *p*(**x***_t_*| **x***_t_*_−1_), should only depend on the previous state estimate and not on the current observations. Therefore, directly incorporating the latest measurements into the particle filter's motion model, is mathematically incorrect and would force us to abandon the Bayesian framework which forms the theoretical foundation of particle filters.

We therefore propose to incorporate the context classification results into the proposal distribution of the particle filter. However, to do so we need to abandon the widely used bootstrap filtering idea (also known as the “condensation” algorithm [43]), in which a particle filter iteration simply corresponds to a prediction step and an update step. Bootstrap filters are probably the most widely used type of particle filter and are often used as a synonym of particle filtering. However, bootstrap filters make an important, yet often invalid assumption to simplify the particle filter theory to a simple set of prediction-update iterations. In the following paragraphs, we review the theoretical foundations of the particle filter in order to show how a custom proposal distribution can improve the tracking results.

Particle filters are used to obtain an estimate of a certain state variable x at each time instance *t* by sequentially integrating new observations into a probabilistic framework. To be able to cope with non-linearities in the observation or motion models, and to deal with non-Gaussian likelihoods, a Monte Carlo simulation is used to directly represent the posterior PDF as a weighted sum of *N* discrete samples (a.k.a. particles) as shown by Equation (5).

(5) $$p\left({\text{x}}_{t}|{\text{y}}_{1:t}\right)\approx \sum_{i=1}^{N}w_{t}^{i}\delta ({\text{x}}_{t}-{\text{x}}_{t}^{i})$$

where ${\text{x}}_{t}^{i}$ is a random sample from this distribution ${\text{x}}_{t}^{i}\sim p\left({\text{x}}_{t}|{\text{y}}_{1:t}\right)$, and $w_{t}^{i}=\frac{1}{N}$. In practice however, drawing samples from the posterior is impossible because the posterior distribution is exactly what we are trying to estimate. On the other hand, for a given observation **y***_t_*, the likelihood $p\left({\text{y}}_{t}|{\text{x}}_{t}^{i}\right)$ can often be easily obtained.

To approximate a distribution that can not be sampled directly but whose likelihood can be evaluated easily if a sample is given, a technique called importance sampling can be used. Importance sampling states that a distribution can be approximated by weighting random samples from any other distribution whose support includes the support of the distribution to be approximated. The distribution from which samples are drawn is called the proposal distribution, and the weights *w^i^* of the drawn samples are obtained as the ratio of the samples' likelihood according to the distribution to be approximated, and their likelihood according to the proposal distribution. The resulting set of weighted samples then represents an approximation of the original distribution.

Particle filters are based on importance sampling as follows: Let the proposal density, also called importance density, be *q*(.). According to Bayes' rule, we can write the posterior to be estimated as *p*(**x***_t_*|**y**_1:_*_t_*) = *αp*(**y**_1:_*_t_*|**x***_t_*) *p*(**x***_t_*), where α is a normalization factor that is equal for all the samples drawn from *q*(.). Based on the concept of importance sampling, the importance weight of each particle can then be calculated as shown by Equation (6).

(6) $$w_{t}^{i}=\frac{p\left({\text{y}}_{1:t}|{\text{x}}_{t}^{i}\right)p\left({\text{x}}_{t}^{i}\right)}{q\left({\text{x}}_{t}^{i}|{\text{y}}_{1:t}\right)}$$

Moreover, under the Markov assumption, these weights can be estimated recursively [44] as

(7) $$w_{t}^{i}=w_{t-1}^{i}\frac{p\left({\text{y}}_{t}|{\text{x}}_{t}^{i}\right)p\left({\text{x}}_{t}^{i}|{\text{x}}_{t-1}^{i}\right)}{q\left({\text{x}}_{t}^{i}|{\text{x}}_{1:t-1}^{i},{\text{y}}_{1:t}\right)}$$

The numerator of Equation (7) is then simply the product of the likelihood (*i.e.*, observation model) $p\left({\text{y}}_{t}|{\text{x}}_{t}^{i}\right)$ and the state transition prior (*i.e.*, motion model) $p\left({\text{x}}_{t}^{i}|{\text{x}}_{t-1}^{i}\right)$. The remaining problem now is how to choose the proposal distribution $q\left({\text{x}}_{t}^{i}|{\text{x}}_{1:t-1}^{i},{\text{y}}_{1:t}\right)$.

In theory, the proposal distribution can be any distribution that includes the support of the posterior. However, the closest the proposal distribution matches the posterior distribution, the more efficient the sampling process is. In practice, choosing a good proposal distribution is often a difficult problem, and most particle filter implementations, such as the well known “condensation” algorithm [43], simply use the state transition prior $p\left({\text{x}}_{t}^{i}|{\text{x}}_{t-1}^{i}\right)$ as the proposal distribution. These type of particle filters are called bootstrap filters and are the most widely used particle filter variant.

A major disadvantage of the bootstrap filter approach however, is that it ignores the fact that the proposal distribution is conditioned on the latest observation. Instead, a bootstrap filter assumes that the current state is only a function of the previous state and is independent of the latest observation. Therefore, bootstrap filters do not fully exploit the power of the Bayesian filtering framework since they assume that the posterior changes smoothly over time and thus closely resembles the transition prior at each time step *t*. Plugging the state transition prior into Equation (7) indeed results in a greatly simplified weight calculation:

(8) $$w_{t}^{i}=w_{t-1}^{i}\frac{p\left({\text{y}}_{t}|{\text{x}}_{t}^{i}\right)p\left({\text{x}}_{t}^{i}|{\text{x}}_{t-1}^{i}\right)}{p\left({\text{x}}_{t}^{i}|{\text{x}}_{t-1}^{i}\right)}=w_{t-1}^{i}p\left({\text{y}}_{t}|{\text{x}}_{t}^{i}\right)$$

**Figure 8.**
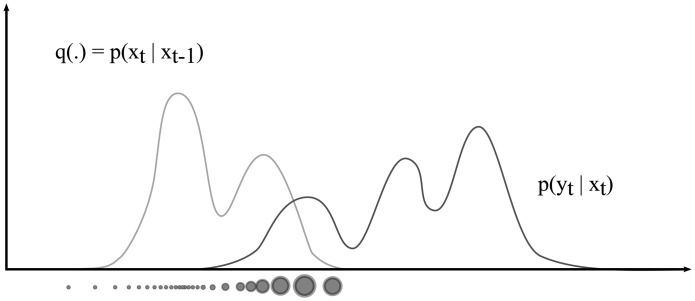
Bootstrap filters use the state transition prior as a proposal distribution. This can cause particle depletion in which few particles are assigned a high weight whereas most particles only model the tails of the posterior.

The proposal distribution is cancelled out in the bootstrap filter equation, and as a result only a prediction and an update needs to be performed at each time instance *t*. Although bootstrap filters are the most widely used type of particle filter, failing to introduce the latest observation into the proposal distribution causes particle depletion if either the state transition prior or observation likelihood has small tails and is highly peaked, or if the state transition prior does not include the support of the posterior. This is illustrated by Figure 8 in which the state transition prior is used as a proposal distribution. Particles are sampled according to the state transition prior, and are then assigned a weight according to the observation likelihood. In this illustration, the size of each particle represents its likelihood. Due to the use of the state transition prior as a proposal distribution, only few particles are assigned a high weight whereas most particles will only model the tails of the posterior.

To avoid this problem, we abandon the bootstrap filter idea, and design a more appropriate proposal distribution based on our context classifier, such that the proposal distribution depends both on the previous state estimate and on the latest measurements. However, it is important to note that the transition prior in the numerator of Equation (7) is no longer cancelled out by the denominator in this case. Therefore, not only do we need an analytic expression of the proposal distribution such that it can be sampled, we also need to calculate the actual transition prior likelihood during weight assignment.

We propose to represent the proposal distribution of each particle by a Kalman filter which incorporates the context classification results into its measurement model. Each particle is then drawn from its corresponding Gaussian distribution, defined by the Kalman filter, during the importance sampling step. Using Equation (7) a weight is then assigned to the sample, based on the particle filter's observation and motion models.

This idea is based on the “Unscented Particle Filter” algorithm, proposed by van der Merwe *et al.* [44]. Their method uses an Unscented Kalman filter [45] to generate a proposal distribution that can be used for non-linear filtering problems. However, the unscented transform is computationally expensive, and unnecessary if a linear motion model is used. In our case, a simple constant velocity and thus linear motion model is employed such that a standard Kalman filter can be used to obtain the proposal distribution. For each particle, the Kalman filter allows us to generate a proposal distribution that incorporates both the context classification results and the constant velocity motion estimate in an optimally weighted manner.

Although Kalman filters assume Gaussian likelihoods and linear models, it is important to note that our particle filter solution, with Gaussian proposal distributions, does not require the posterior to be normally distributed and does not require linear observation models. The reason for this is that a distinct Kalman filter is used per individual particle, from which the new particle state will be sampled during the importance sampling process. Hence only the distribution of each single particle is assumed to be Gaussian. During importance sampling, the particle is drawn from the normal distribution that serves as the proposal distribution, as shown by Equation (9).

(9) $$\begin{array}{cc} q\left({\text{x}}_{t}^{i}|{\text{x}}_{t-1}^{i},{\text{y}}_{1:t}\right)∼N\left({\bar{\text{x}}}_{t}^{i},P_{t}^{i}\right) & i=1,\ldots ,N \end{array}$$

where ${\bar{\text{x}}}_{t}^{i}$ represents the Kalman filter's state estimate and $P_{t}^{i}$ represents its covariance matrix.

Thus, at time *t*, the latest observation **ŷ**, defined by Equation (4), is used to obtain the mean, ${\bar{\text{x}}}_{t}^{i}$, and covariance matrix $P_{t}^{i}$ of the proposal distribution of each particle *i*, using the Kalman filter measurement and update equations. Next, the *i^th^* particle is sampled from this distribution, and is assigned a weight by the observation model, as in a traditional particle filter. This method requires us to propagate the covariance $P_{t}^{i}$ for each particle since multiple particles might share the same covariance matrix after resampling.

In the following, let **x**(*t* |*t* − 1) be the state estimate at time instance *t*, given observations up to and including time *t* − 1. We need to define the motion model matrix *A* that is used to predict the next state based on the previous state estimate as **x**(*t* |*t* − 1) = *A* **x**(*t* − 1|*t* − 1) + **m**, where **m** is the noise component. In our case, *A* corresponds to the 4 × 4 matrix that represent the following constant velocity transformation:

(10) $${\text{x}}_{t}=2{\text{x}}_{t-1}-{\text{x}}_{t-2}$$

(11) $$={\text{x}}_{t-1}+({\text{x}}_{t-1}-{\text{x}}_{t-2})$$

(12) $$={\text{x}}_{t-1}+\Delta V$$

where Δ*V* models the velocity of the object in the previous video frame. For state vector **x***_t_* = (*x_t_*, *y_t_*, *x_t_*_−1_, *y_t_*_−1_), the motion model matrix *A* then becomes:

(13) $$A=\left[\begin{array}{llll} 2 & 0 & -1 & 0\\ 0 & 2 & 0 & -1\\ 1 & 0 & 0 & 0\\ 0 & 1 & 0 & 0 \end{array}\right]\;,{\text{x}}_{t-1}=\left[\begin{array}{l} x_{t-1}\\ y_{t-1}\\ x_{t-2}\\ y_{t-2} \end{array}\right]$$

Similarly, the observation matrix *H* needs to be defined, such that **ŷ** = *H* **x**(*t*|*t* − 1) + **n**, where **n** is a white noise component and where **ŷ** is the context classifier based observation defined by Equation (4). Since both the state estimate x and the observation **ŷ** consists of a coordinate pair (*x*, *y*) representing the hand location, the observation matrix *H* simply reduces to:

(14) $$H=\left[\begin{array}{llll} 1 & 0 & 0 & 0\\ 0 & 1 & 0 & 0 \end{array}\right]$$

In the following, let *Q* be the covariance matrix that defines the white Gaussian process noise component **m**, and let *R* be the covariance matrix that defined the white Gaussian measurement noise component **n**. For each particle *i* in the set of *N* particles, the Kalman filter estimates the mean *μ^i^* = **x**(*t*|*t*) and covariance matrix *P^i^* = *P*(*t*|*t*) of the multivariate Gaussian proposal distribution *q*(.) ∼ *N*(*μ^i^*, *P^i^*). The state prediction at time *t*, given observations up to and including time *t* − 1 is:

(15) x(t|t−1)=Ax(t−1|t−1)

and the corresponding covariance is

(16) $$P(t|t-1)=AP(t-1|t-1)A^{\text{T}}+Q$$

The new estimate, after incorporating the context classification result **ŷ**, is then:

(17) $$\text{x}(t|t)=\text{x}(t|t-1)+K(t)[\hat{\text{y}}(t)-H\text{x}(t|t-1)]$$

with covariance

(18) $$P(t|t)=P(t|t-1)-K(t)S(t)K{\left(t\right)}^{\text{T}}$$

where the Kalman gain is

(19) $$K=P(t|t-1)H^{\text{T}}S^{-1}(t)$$

and the innovation covariance is

(20) $$S(t)=HP(t|t-1)H^{\text{T}}+R$$

If *N* particles are used to represent the posterior distribution, then also *N* Kalman filters are used to generate particle-specific proposal distributions. The particle filter then draws *N* new particles, each from their corresponding proposal distribution. In the following, let ${\text{x}}_{t}^{i}$ be the state of the particle that is sampled from this proposal distribution of particle *i* at time instance *t*. Let ${\dot{\text{x}}}_{t}^{i}$ be the state estimate of the same particle, obtained by the motion model of the particle filter. The state transition prior for this particle *i* is then obtained by evaluating the likelihood of the sample ${\text{x}}_{t}^{i}$ by the particle filter's motion model. If the motion model's likelihood function is normally distributed, centered at the state that was predicted by the constant velocity model, then the state transition prior is:

(21) $$p({\text{x}}_{t}^{i}|{\text{x}}_{t-1}^{i})\sim e^{-\frac{1}{2}{\left({\text{x}}_{t}^{i}-{\dot{\text{x}}}_{t}^{i}\right)}^{\text{T}}Q^{-1}({\text{x}}_{t}^{i}-{\dot{\text{x}}}_{t}^{i})}$$

The above equation shows that, although we do not assume a linear observation model for the particle filter, and we do not assume a Gaussian posterior distribution, we do assume a Gaussian state transition prior and a linear motion model. However, most particle filter implementations use simple linear motion models like the constant velocity or the random walk model, such that this constraint does not pose a practical problem.

The proposal density likelihood for sample *i* is defined by the Gaussian posterior that is estimated by the Kalman filter:

(22) $$q({\text{x}}_{t}^{i}|{\text{x}}_{1:t-1}^{i},{\hat{\text{y}}}_{1:t})\sim \frac{1}{\sqrt{\left|P_{t}^{i}\right|}}e^{-\frac{1}{2}{\left({\text{x}}_{t}^{i}-\mu_{t}^{i}\right)}^{\text{T}}{\left(P_{t}^{i}\right)}^{-1}({\text{x}}_{t}^{i}-\mu_{t}^{i})}$$

Finally, the observation likelihood $p\left({\text{y}}_{t}|{\text{x}}_{t}^{i}\right)$ is obtained using the skin-color and motion based measurement model of our particle filter as discussed in [16]. Substituting Equations (21), (22) and the observation likelihood into Equation (7) allows us to calculate the particle weights $w_{t}^{i}$ as follows:

(23) wti=wt−1ip(yt|xti)p(xti|xt−1i)q(xti|x1:t−1i,y^1:t)

In contrast to the widely used bootstrap filter, our approach is able to incorporate the latest observations into the proposal distribution to greatly improve sampling efficiency. As a result, relatively few particles (50 in our experiments) are needed for robust tracking. Furthermore, since the proposal density is based on the context classification result, particles are samples around the maxima of the cross-correlation map shown by Figure 7 and are therefore less sensitive to distractions caused by hand-like objects in the neighborhood.

### 3.4. Context Based Arm Tracking Using Partitioned Sampling

In the previous section we proposed a method to incorporate contextual information into a particle filter tracking framework. Contextual information in this setting could be the arm if tracking a hand, but might as well be a shadow when tracking vehicles [46] or a limb when tracking articulated objects in general.

In this section we introduce a second method to improve tracking performance. This solution however is specific to hand tracking, and improves robustness against occlusion by explicitly tracking the arm. In the previous section, the state vector was defined as **x** = (*x*, *y*) and represented the coordinates of the hand to be estimated. We now extend the state vector with a parameter that describes the angle *θ* of the arm. The state vector then becomes **x** = (*x*, *y*, *θ*).

Directly estimating the joint probability *p*(*x*, *y*, *θ*|**y**_1:_*_t_*) would require exponentially more particles to be used compared to estimating the previous posterior *p*(*x*, *y*|**y**_1:_*_t_*). Intuitively this can be explained as follows: If 10 samples would be needed to sample a 1-dimensional unit interval with sufficient sample density, then 10^2^ = 100 samples would be needed to model a two-dimensional problem with the same density, and 10^3^ = 1000 samples would be needed in the 3D case. In the above problem, 50 particles are used such that ${\left(\sqrt{50}\right)}^{3}=354$ particles would be needed to maintain the same sample density in the current 3D state space. However, this would greatly increase the computational complexity of the particle filter, preventing real-time operation.

To avoid the curse of dimensionality, we partitioned our search space based on the assumption that the arm angle is independent of the (*x*, *y*) location of the hand. Particle filtering in a partitioned state space is called partitioned sampling and was proposed by MacCormick *et al.* [47]. They showed that different state partitions can be solved for sequentially instead of simultaneously if a hierarchical relation exists between the subspaces. We use this concept to transform our 3D search space to a sequence of a 2D and a 1D estimation problem.

Partitioned sampling can be considered the statistical equivalent of a hierarchical search, in which the child node in a tree depends on the state of its parent. In the current setting, we first estimate the hand coordinates, and use this estimate to obtain an estimate of the arm angle if contextual information is available. An additional advantage of this approach is that the second partition, *i.e.*, estimating *θ*, can easily be omitted if no contextual information is available at a time instance *t*.

**Figure 9.**
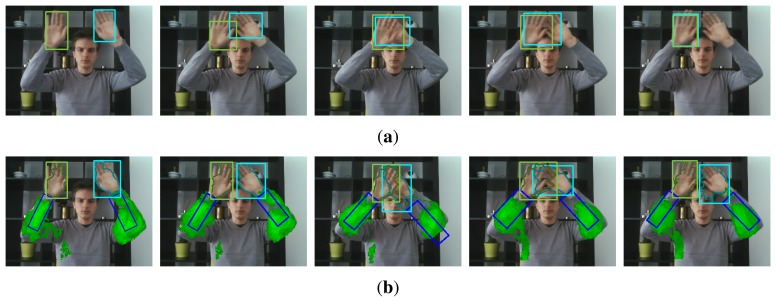
Top row: Hand tracking fails due to occlusion. Bottom row: Arm tracking helps to disambiguate in case of clutter or occlusion. **(a)** Hand tracking as proposed in [16]. **(b)** Arm and hand tracking using partitioned sampling.

A valid question is now how estimating *θ* would improve the estimate of the hand location (*x*, *y*), since the hand location is estimated before and independently from the arm angle. However, it is important to note that particle filters employ a resampling strategy to avoid the particle depletion problem. When the variance of the particle weights becomes large, the sample set is replaced by a new sample set by resampling with replacement. This means that particles with a bad *θ* estimate and corresponding low arm-likelihood, get replaced by duplicates of particles with a good *θ* estimate with corresponding high arm-likelihood. In the next iteration, the (*x*, *y*) location of the hand is then estimated for this new set of particles. Therefore, arm tracking makes sure that particles with a high skin-color and motion likelihood are deleted from the sample set if they correspond to a low arm-likelihood. Furthermore, resampling only occurs if the variance of the particle weights becomes large and thus when the covariance of the particles increases. Therefore, the correcting effect of the arm tracking partition is negligible during stable hand tracking (almost no resampling occurs), whereas it becomes more apparent in case of clutter, and occlusion (resampling occurs often). This is illustrated in Figure 9, where the top row shows the tracking result if only the first partitioned is estimated, whereas the bottom row shows the result if also the arm angle is tracked.

Whereas a custom proposal distribution was used to estimate the parameters of the first state partition, *i.e.*, the hand location, a simple bootstrap filter was used for the second partition, *i.e.*, the arm angle. The state transition prior is therefore used as a proposal distribution, and the motion model for this partition is a random walk model.

Our choice of observation model used to obtain the likelihood for each estimate *θ_i_* of the arm location is again driven by the real-time constraints of our tracking solution and is therefore computationally inexpensive. For each particle, the arm is represented by a straight line segment, the length of which is proportional to max(*w*, *h*), where (*w*, *h*) defines the width and height of the hand's bounding box. Each point on this line corresponds to a context probability *p*(*l* = 1|*x*, *y*), indicated in green in Figure 9. We obtain the probability that a line represents the arm by marginalizing this conditional density such that *p*(*l* = 1;θ) = Σ*_x,y_ p*(*l* = 1|*x*,*y*; *θ*), where *θ* is the arm angle that defines which points (*x*, *y*) are considered. The actual arm angle then corresponds to

$$\hat{\theta }=\underset{\theta }{\arg\max}p(l=1;\theta )$$

**Figure 10.**
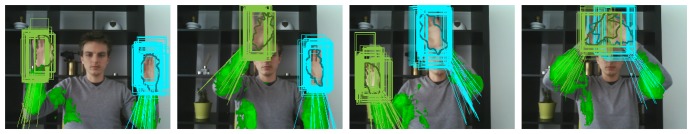
Visual representation of the particle filter's posterior distribution. The (*x*, *y*, *width*, *height*) parameters of the particles are represented by colored rectangles, whereas the arm angle *θ* is represented by line segments.

Iterating over all pixel coordinates (*x*, *y*) defined by the line segment with angle *θ* can be implemented extremely efficiently using the well known Bresenham line algorithm. Figure 10 shows all particles of each particle filter visually for illustrative purposes.

## 4. Results and Discussion

In this section, we compare our hand tracking results with several state-of-the-art tracking algorithms using three publicly available datasets. The first dataset was created to specifically test tracking robustness against occlusion and cluttered backgrounds. This dataset contains eight video sequences with an average duration of 45 s, and is made publicly available online (http://telin.ugent.be/vspruyt/sensors/) for research purposes to allow for fair comparison with future methods. The sequences contain cluttered backgrounds, fast motion, both long and short sleeved arms, and several cases of occlusion. Table 1 lists the characteristics of each video in this dataset.

To evaluate the increase in robustness by incorporating contextual information into the original hand tracking method described in [15], we simply count the number of tracking errors in each video sequence. A tracking result is considered erroneous if the Pascal VOC score [48] is below 0.5. The VOC score defines the amount of overlap between the detected bounding box, and the given ground truth bounding box and is calculated as $\text{VOC}=\frac{B_{g}\cap B_{d}}{B_{g}\cup B_{d}}$, where *B_g_* represents the pixels within the ground truth bounding box, whereas *B_d_* represents the pixels within the detected bounding box.

Table 2 compares the number of tracking errors for the original algorithm of [15] with the number of tracking errors obtained using the approach proposed in this paper based on the new proposal distribution and our arm tracking solution.

These results illustrate that both methods proposed in this paper to incorporate contextual information into the tracking framework contribute to an increased tracking robustness. Both methods are complementary as can be seen by the decrease in tracking errors when combined, e.g., in video 2, video 4, video 7 and video 8.

**Figure 11.**
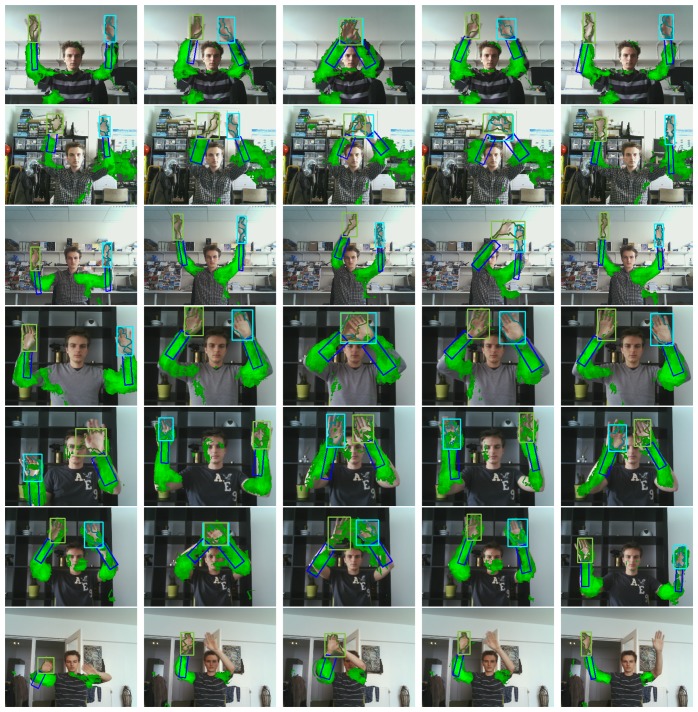
Several hand tracking results for the newly proposed publicly available dataset. Each row corresponds to a different video sequence in this dataset.

The first six video sequences are meant to test the tracker's robustness against occlusion. Results for these sequences are shown by Figure 11.

**Figure 12.**
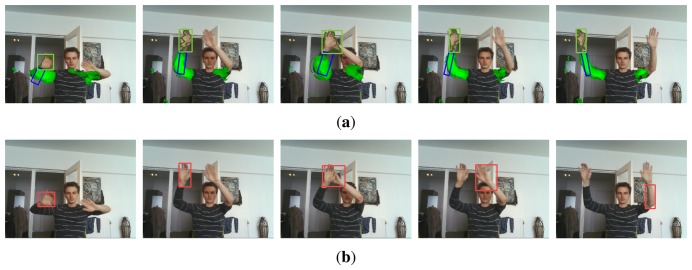
Several video frames from sequence 7, in which only the left hand is tracked such that the right hand can serve as a distraction to test robustness against occlusion with unknown hand-like objects. (**a**) Our result (proposal + arm tracking). (**b**) Result of [15].

Video 7 serves as a special case. In this sequence we deliberately tracked only one hand, such that the other hand and its corresponding arm can serve as a distraction as if it was any moving, hand-like object. This allows us to evaluate the behaviour of the tracking algorithm in case of occlusion with unknown, hand-like objects. Figure 12 compares the tracking results of this sequence by the algorithm described in [15] with the results obtained using the proposed method.

These results clearly show the benefits of incorporating contextual information into the tracking process. Whereas the original hand tracking algorithm is unable to distinguish between the tracked left hand and the unknown right hand, context learning allows the particle filter to use spatial dependencies to resolve ambiguous situations.

**Figure 13.**
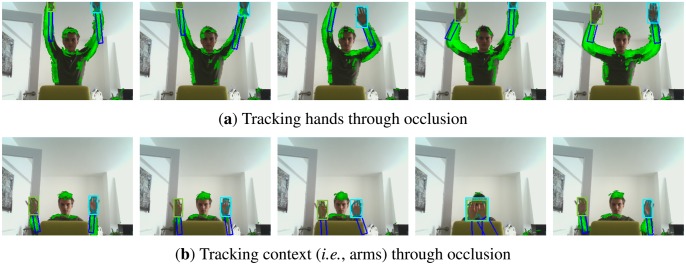
Several video frames from sequence 8, in which both the hands (**a**) and the arms (**b**) are completely occluded. Incorporating context in the proposal distribution resolves ambiguity when the object of interest is occluded. On the other hand, if the context itself is occluded, the proposal distribution becomes non-informative and does not hurt tracking performance. The predicted arm location in this case still increases robustness of the hand tracker.

Video 8 contains several cases of severe occlusion of both the object of interest, *i.e.*, the hand moves out of picture, and its context, *i.e.*, the arm moves behind a chair, as shown in Figure 13. When the hand is completely occluded, the context based proposal distribution allows the particle filter to overcome the local minimum of the observation likelihood, thereby increasing tracking robustness. When the context itself is completely occluded, it does not contribute to the proposal distribution, such that the particle filter falls back to its normal bootstrap filter behavior. However, the partitioned sampling based arm tracking stage still predicts the most likely position of the arm, which in turn helps the hand tracking stage to overcome ambiguity due to occlusion between the left and right hands.

**Figure 14.**
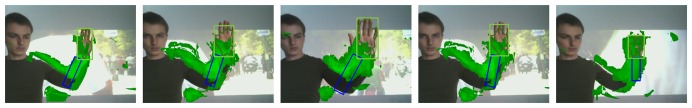
Several video frames from sequence 9, in which the hand only moves perpendicular to the camera plane while background motion serves to test the tracker's robustness.

Sequence 9 contains a lot of background motion. The video sequence was captured while projecting a movie clip on the background wall. The hand only moves perpendicular to the camera plane, *i.e.*, towards and away from the camera. Therefore, this sequence challenges the capabilities of the context learning method which depends on optical flow similarity between the tracked hand and its context. Figure 14 shows several frames from sequence 9. Although the context segmentation is less accurate than in the other videos, due to the challenging background motion, the results illustrate the robustness of our method in unconstrained environments.

**Figure 15.**
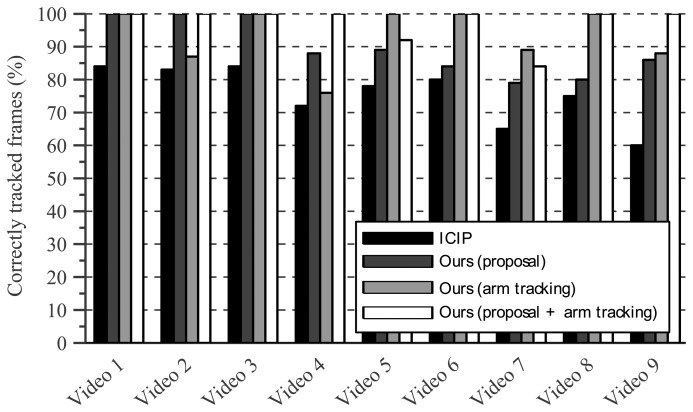
Percentage of correctly tracked frames in each video sequence. When tracking failure occurs (Table 2), all frames are considered incorrectly tracked until the VOC score exceeds 0.5 again.

Figure 15 shows the percentage of frames that is tracked correctly in each video sequence. Whereas Table 2 listed the number of times a tracking error begins, Figure 15 also incorporates the duration of each tracking failure by reporting the fraction of video frames that was tracked correctly.

**Figure 16.**
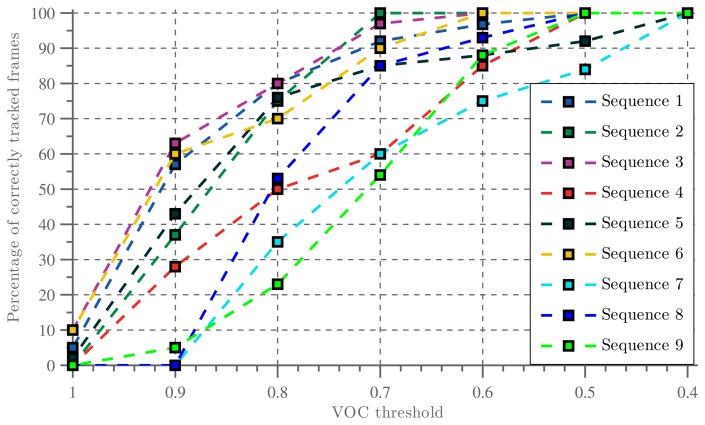
Percentage of correctly tracked frames in each video sequence, plotted against the VOC score. Perfect tracking is obtained if results with a VOC score of 0.4 and higher are considered to be correctly tracked instances. A VOC threshold of 0.5 is often used in object detection research to indicate correct detections.

These results consider tracking to be failed when the resulting bounding box corresponds to a VOC score lower than 0.5. However, many practical applications would benefit from a lower threshold, since even a VOC score of 0.2 corresponds to a reasonable tracking results. Therefore, Figure 16 shows the percentage of correctly tracked video frames, plotted against the corresponding VOC score. A VOC threshold of 0.5 is often used in object detection research to indicate correct detections, whereas a VOC threshold of 0.25 is common in face detection literature.

Although the focus of this paper is context based hand tracking, we showed that the context can be used to obtain an estimate of the arm location. To evaluate the arm tracking capabilities, we compared our method with the pictorial structure based upper body pose detector described by Eichner *et al.* [49]. This method estimates the spatial structure of the upper body. Although no temporal information is used by their method, the algorithm requires a bounding box of the upper body as input and can therefore be compared fairly with our tracking algorithm.

**Figure 17.**
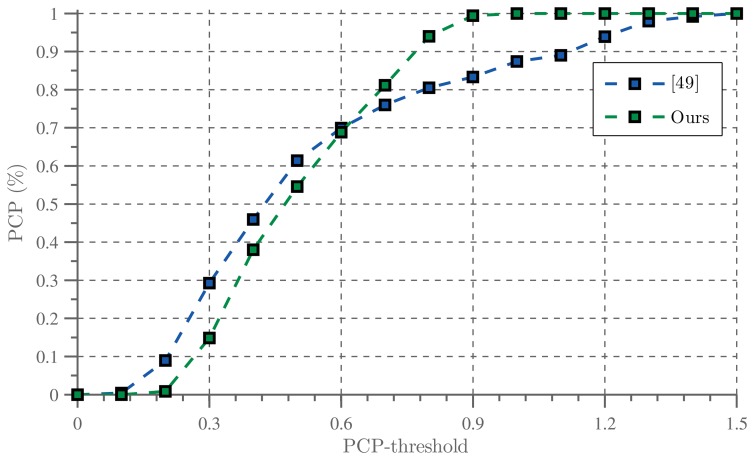
Comparison of the arm tracking results of our method with the results obtained with the pictorial structure based algorithm proposed by [49]. The PCP score represents the percentage of correctly tracked arm instances, whereas the PCP-threshold defines when a detected instance is considered correct.

In [49], the algorithm is evaluated by reporting the Percentage of correctly estimated body Parts (PCP). An estimated body part, *i.e*., the arm, is considered correct if its segment endpoints lie within a fraction *f* of the length of the ground-truth segment. In the following, we adopt this evaluation measure and compare their method with ours by reporting the PCP while varying the threshold *f* ∈ (0, 1.5). The results are illustrated by Figure 17.

Although our method appears slightly less accurate when the PCP threshold is low, its accuracy increases rapidly and outperforms [49] for higher PCP thresholds. Our method is able to provide a rough estimate of the arm pose, even in cluttered scenes with complicated interactions, whereas the algorithm proposed by [49] provides an accurate estimate in simple sequences but quickly breaks down in cluttered scenes. Furthermore, [49] requires approximately 1.5 s of processing time per video frame, whereas our solution processes 17 frames per second.

**Figure 18.**
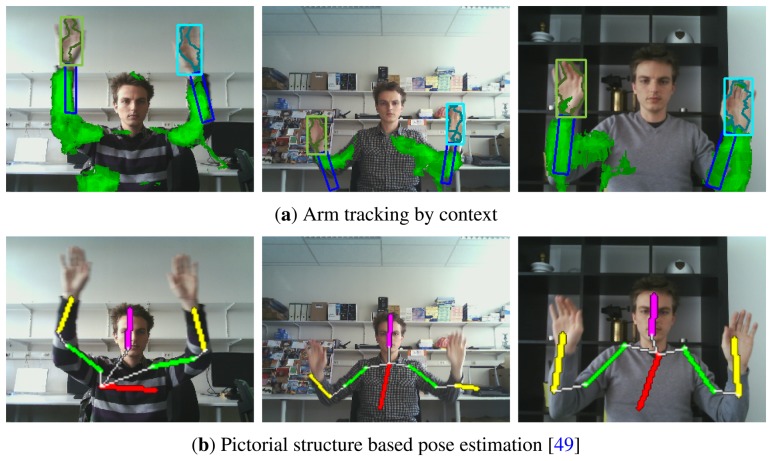
Some example frames comparing our arm tracking method with the pictorial structure based algorithm proposed by [49].

Figure 18 illustrates some of the results obtained by both methods.

Finally, Figure 11 shows several results on the proposed dataset for illustrative purposes.

Whereas evaluation results on this challenging dataset clearly indicate the advantages of our solution, a second question of interest is whether or not context learning would degrade tracking performance in the cases where near-perfect tracking can already be accomplished without contextual information.

To evaluate this question, we performed additional tests using the publicly available dataset from [15]. This dataset contains eight video sequences each spanning approximately 90 s. These sequences contain changing illumination, fast motion, camera motion, and both long and short sleeved arms. The dataset was proposed in [15] and is publicly available for academic research, together with manually annotated groundtruth bounding boxes for both hands. Table 3 summarizes the characteristic of each video sequence in this dataset.

As listed by Table 3, this dataset does not contain occlusions. Even for sequence 8, the occlusions that occur are partial occlusions behind semi-transparent objects. The hands never completely overlap in these sequences, whereas the major novelty introduced in this paper specifically increases robustness in case of occlusion. Furthermore, the results obtained by the original hand tracker upon which the work presented here is based, are already impressive; yielding an average VOC score of 76%. This means that the tracker of [15] never looses track of the target object and is able to accurately model its appearance at all times.

**Figure 19.**
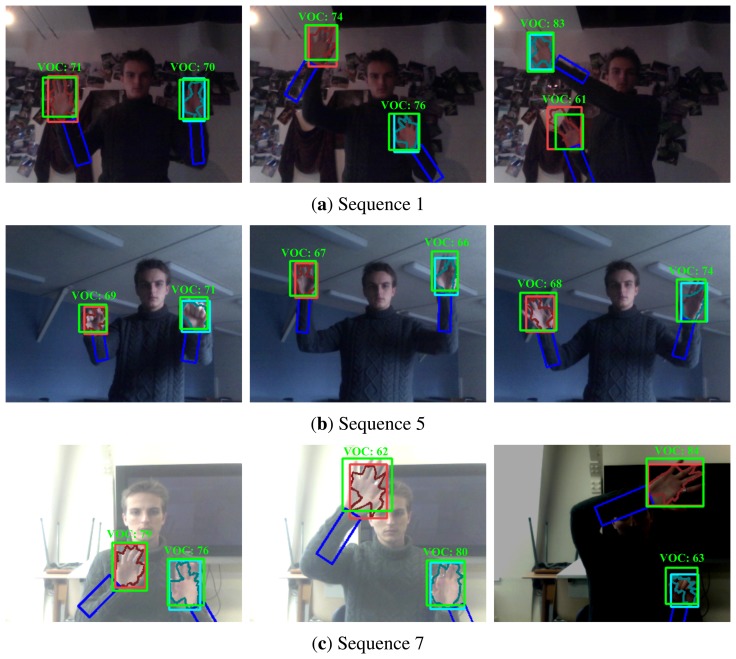
Several hand tracking results for the publicly available dataset from [15] with corresponding VOC score (expressed as a percentage). The groundtruth is indicated by a green rectangle. VOC scores higher than 0.5 are usually considered correct detections in object detection literature.

Figure 19 shows the VOC scores obtained on these sequences using the method proposed in this paper. These results clearly show that a VOC score around 75% represents almost perfect tracking in the sense that higher VOC scores would probably not be very meaningful because even the groundtruth annotations might not be that perfect. Therefore it is obvious that no tracker could largely outperform the near perfect results reported in [15] on this dataset. Nevertheless, it would be interesting to see if the addition of contextual information would degrade the results in this case, especially since this dataset contains abruptly changing illumination, moving camera's and very fast motion.

**Figure 20.**
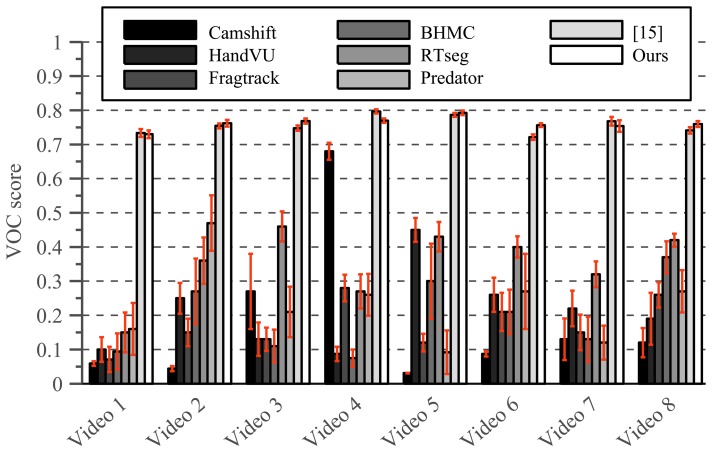
VOC score comparison for several state-of-the-art tracking algorithms on the publicly available hand dataset from [15].

Figure 20 shows the VOC scores for several state-of-the-art algorithms, applied to this dataset. Each method was initialized manually based on the ground truth bounding boxes in the first video frame to allow for a fair comparison. The first method is the Camshift algorithm [50] which is often used as a baseline when evaluating tracking methods in computer vision. The second method is the HandVU system [9], a well known hand-tracking method based on a combination of color cues and Harris corner features that are spatially constrained using flocking behaviour rules. Third, we compare our method with the RTseg solution that we proposed earlier in [14]. The RTseg method combines multiple color spaces using a Gaussian Mixture Model, in an attempt to decrease illumination dependence. The fourth algorithm is the Predator tracker [23] which was discussed in Section 2 and is a patch-based tracking solution that employs PN-learning to automatically learn the object's appearance over time. Whereas the predator tracker is designed to track rigid objects and is therefore expected to fail when the hand undergoes large deformations, the adaptive basin hopping monte carlo filter (BHMC) proposed in [51] is an adaptive patch based tracker designed for tracking non-rigid objects. Finally, the Fragtrack algorithm [52] implements the generalized Hough transform to perform patch based tracking and uses integral histograms to efficiently represent the object's appearance. The method labelled [15], is the hand tracking algorithm we proposed in [15] and forms the base of the solution described in this paper.

This method use a combination of color and motion cues, optical flow and a discriminative Hough voting scheme.

These results show that our solution maintains the near perfect tracking results on this dataset, with the added value that the location and a segmentation of the arm is obtained automatically.

Finally, similar to the HandVU and Predator algorithms, our non-optimized C++ code processes QVGA data in 70 milliseconds, resulting in a framerate of 14 fps on a quadcore Intel I7 architecture with 8 gigabytes of RAM. The original hand tracking algorithm proposed in [15] runs at 18 fps such that the overhead, introduced by the contextual learning, is negligible compared to the benefit of increased robustness. As a comparison, both the BHMC and the Fragtrack algorithms need several seconds per video frame and are unable to robustly track hands.

Finally, it would be interesting to compare our hand tracking results with tracking algorithms that employ pictorial structure to track articulated objects. To this end, we compared our method with two well know upper body tracking solutions [53,54], applied to the publicly available and widely used Signers dataset [55]. The Signers dataset contains five real and challenging BBC news sequences with signers performing a sign language translation.

Pictorial structure based approaches employ prior knowledge about the articulated nature of an object of interest to infer its joint configuration. Buehler *et al.* [53] proposed an efficient method to sample from a pictorial picture proposal distribution to perform upper body tracking. Their method searches for key frames in which accurate detection can be achieved, and performs temporal tracking between these key frames based on HOG features and color cues. Their solution is able to accurately detect and track upper and lower arms, and uses this information to infer the hand location.

Buehler *et al.* [53] report the percentage of video frames in which hands were correctly tracked. Hands are considered to be tracked correctly, if the VOC score is above a certain threshold. Results are reported for VOC scores of *VOC* ≥ 0.2, *VOC* ≥ 0.5 and *VOC* ≥ 0.6. Table 4 compares the results obtained by Buehler *et al.* with the results obtained by [15] and with the results obtained by the method proposed in this paper.

These results show that the incorporation of context clearly improves tracking performance for this real-life dataset, when compared to the original tracking method, proposed in [15]. This can be explained by observing the first row of Table 4: Without context based information, the target hand is lost during tracking several times. When context is added however, tracking accuracy still varies, but the hand is never truly lost.

Furthermore, our method provides a higher precision (row two of Table 4) than the method proposed by Buehler *et al.*, when applied to the task of hand tracking. On the other hand, Buehler's approach yields a slightly higher accuracy (last row of Table 4) than ours, and has the added advantage of returning an accurate estimate of both the upper and lower arm position. However, their approach requires approximately 100 s of processing per video frame, whereas our proposed solution operates in real-time, processing approximately 17 frames per second.

A related, pictorial structure based approach was proposed by Karlinsky *et al.* [54], who detect hand and arm configurations using an ensemble of feature chains leading from the face location to the hand location. In their method, a face detector is used to estimate the face location, after which the chains model infers the upper body configuration. Karlinsky *et al.* assume the hand width equal to half the face size, and consider a hand detection to be correct if it is within one hand width from the ground truth location.

Finally, Kumar *et al.* [56] proposed a discriminative parts based classifier to detect the upper body of a person by embedding pictorial structure. Although their method performs object detection and therefore does not incorporate temporal information, we include their results on the Signers dataset for completeness.

Table 5 shows the results for both methods on the Signer dataset, compared to the results obtained by our method with and without contextual information.

Although the chains model proposed by Karlinsky does not employ temporal information, it does require the face location to be known for initialization purposes in each video frame. As such, it can be compared fairly to tracking algorithms. The results illustrated by Table 5 show the advantage of incorporating contextual information into a tracking framework, considering the real-time performance of our solution whereas the pictorial structure based approach of Kumar *et al.* needs approximately 15 s of processing time per video frame.

**Figure 21.**
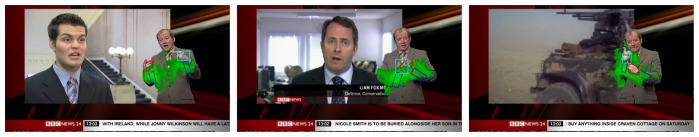
Illustration of hand tracking on the Signer dataset. Although context modeling is not perfect due to the uniform color of arms and body of the signer, the tracker clearly benefits from the local contextual information.

Figure 21 shows several frames from the Signer data set for illustrative purposes.

## 5. Conclusions

In this paper we proposed a novel method to automatically learn the context of a tracked object in an unsupervised manner. Furthermore, we introduced two approaches to incorporate this information into a particle filter tracking framework to improve the tracker's robustness against occlusion and distracting background objects. Our algorithm operates in real-time, and as a by-product can output a rough segmentation of the object's context, *i.e.*, the arm in the case of hand tracking. We showed that our solution outperforms the state-of-the-art when dealing with occlusions, whereas tracker performance is not degraded when no occlusions are present. Finally, our method runs in real-time, processing approximately 14 frames per second on modern hardware.

## Figures and Tables

**Table 1.** Test dataset specification.

	Seq. 1	Seq. 2	Seq. 3	Seq. 4	Seq. 5	Seq. 6	Seq. 7	Seq. 8	Seq. 9
Scene clutter	no	yes	yes	yes	yes	yes	yes	no	yes
Fast motion	no	yes	no	no	yes	yes	no	no	no
Short sleeves	no	no	no	no	yes	yes	no	no	no
Hand occlusion	yes	yes	yes	yes	yes	yes	yes	yes	yes
Face occlusion	yes	yes	yes	yes	yes	yes	yes	yes	no
Arm hidden	no	no	no	no	no	no	no	yes	no
Hand hidden	no	no	no	no	no	no	no	yes	no
Textured clothing	yes	yes	yes	no	no	no	yes	no	no
Background motion	no	no	no	no	no	no	no	no	yes
Length	34 s	46 s	67 s	35 s	35 s	56 s	47 s	45 s	45 s

**Table 2.** Evaluation results on the novel dataset. For each algorithm, the number of tracking errors is reported. Both the custom proposal distribution and the partitioned sampling based arm tracking reduce the number of tracking errors on average.

Sequence #	[15]	Ours (Proposal)	Ours (Arm Tracking)	Ours (Proposal + Arm Tracking)
Video 1	2	0	0	**0**
Video 2	1	0	1	**0**
Video 3	1	0	0	**0**
Video 4	4	1	2	**0**
Video 5	3	1	0	**1**
Video 6	2	1	0	**0**
Video 7	6	3	1	**1**
Video 8	4	2	0	**0**
Video 9	6	1	1	**0**

**Table 3.** Test dataset ([15]) specification.

#	Scene	Lighting	Motion	Sleeves	Camera	Occlusion	Length
1	cluttered	dark	normal	long	fixed	no	83 s
2	cluttered	bright	normal	long	fixed	no	82 s
3	normal	normal	fast	long	fixed	no	85 s
4	normal	bright	normal	long	moving	no	88 s
5	normal	normal	normal	long	fixed	no	89 s
6	cluttered	normal	normal	short	fixed	no	84 s
7	normal	changing	normal	long	fixed	no	85 s
8	normal	normal	normal	short	fixed	yes	83 s

**Table 4.** Results on the Signer dataset, reported as the percentage of video frames for which the VOC score exceeds a predefined threshold.

VOC Threshold	Buehler [53]	Spruyt [15]	Our Method
*VOC* ≥ 0.2	**100.0%**	96.0%	**100.0%**
*VOC* ≥ 0.5	94.3%	91.9%	**96.8%**
*VOC* ≥ 0.6	**83.4%**	75.8%	82.6%

**Table 5.** Results on the Signer dataset, reported as the percentage of video frames for which the detected hand location is less than one hand-width from the ground truth location.

Karlinsky [54]	Kumar [56]	Spruyt [15]	Our Method
84.9 %	78.95%	97.3%	**100.0%**
